# Probiotic and Prebiotic Supplementation for Gastrointestinal Discomfort in Chronic Spinal Cord Injury (PRO-GIDSCI): A Randomized Controlled Crossover Trial Protocol

**DOI:** 10.3390/mps9010014

**Published:** 2026-01-17

**Authors:** Julia Trunz, Cyra Schmandt, Anneke Hertig-Godeschalk, Marija Glisic, Jivko Stoyanov, Claudio Perret

**Affiliations:** 1 Swiss Paraplegic Research, 6207 Nottwil, Switzerland; cyra.schmandt@paraplegie.ch (C.S.); anneke.hertig@paraplegie.ch (A.H.-G.); marija.glisic@paraplegie.ch (M.G.); jivko.stoyanov@paraplegie.ch (J.S.); claudio.perret@paraplegie.ch (C.P.); 2 Faculty of Health Sciences and Medicine, University of Lucerne, 6005 Lucerne, Switzerland; 3 Graduate School for Cellular and Biomedical Sciences, University of Bern, 3012 Bern, Switzerland; 4 Institute of Social and Preventive Medicine (ISPM), University of Bern, 3012 Bern, Switzerland

**Keywords:** paraplegia, tetraplegia, gastrointestinal transit time, incontinence, bowel management

## Abstract

Background: Gastrointestinal discomfort affects up to 70% of individuals with spinal cord injury (SCI), largely due to gut dysbiosis caused by altered transit time and reduced gastrointestinal motility from autonomic disruption. Emerging evidence links prebiotics and probiotics to improved microbiome balance and reduced inflammation, yet data in SCI remain limited. Methods: Individuals aged ≥ 18 years, with a chronic SCI (≥1 year) experiencing significant gastrointestinal symptoms, will be invited to participate in this single-center randomized controlled crossover trial. Persons currently taking antibiotics, who have relevant eating or digestive disorders, or who have undergone a recent diet change will be excluded from the study. Participants will be randomized (1:1) into two groups. The first group will take a probiotic (Biotics-G, Burgerstein AG, Rapperswil-Jona, Switzerland) supplement for eight weeks, then after a four-week washout period, they will take a prebiotic (Oat Bran, Naturaplan, manufactured by Swissmill, Zurich, Switzerland) supplement for another eight weeks. The second group will receive the supplements in reverse order. The primary outcome is the Gastrointestinal Quality of Life Index, a questionnaire to assess quality of life related to gastrointestinal disorders. Secondary outcomes consist of gastrointestinal transit time, inflammatory blood markers, and gut microbiome composition. Ethics: The study will be conducted in accordance with the Declaration of Helsinki. The study was approved by the Ethics Committee for Northwest/Central Switzerland (EKNZ, ID: 2025-00238, 24.02.2025, Version 2.0). The study is registered at ClinicalTrials.gov (ID: NCT06870331, 02.04.2025). Written informed consent will be obtained from all participants involved in the study.

## 1. Introduction

In some countries, it has been reported that up to 70% of individuals with a spinal cord injury (SCI) suffer from gastrointestinal (GI) symptoms [1,2,3]. Symptoms like fecal incontinence, abdominal bloating, constipation, and irritable bowel syndrome can substantially compromise quality of life by limiting social participation and psychological well-being. Consequently, previous research has found bowel management to be a top priority among individuals with SCI [4,5]. Bowel management is crucial in recovery and rehabilitation as it majorly affects autonomy and dignity [4,6].

SCI disrupts the bidirectional communication of the central and enteric nervous system, which impairs motor and autonomic output but also disturbs sensory feedback and neuroimmune signaling, all of which contribute to altered enteric nervous system activity [7].

Disruption of neural pathways and autonomic imbalance after the onset of SCI can lead to impaired colonic motility, delayed colonic transit time, and increased barrier permeability in the intestine [8,9,10,11]. These factors contribute to gut dysbiosis and intestinal inflammation, which is increasingly recognized as both a consequence of and contributor to GI complications in SCI [12,13,14]. GI homeostasis, including digestion, nutrient absorption, immune modulation, and barrier integrity, is dependent on the gut microbiome composition [15,16]. A systematic review has recently shown differences in gut microbiota composition in individuals with various GI diseases compared to individuals without GI diseases, which manifest as reduced beneficial bacteria and increased inflammation-associated genera [17]. Gut-derived inflammation may add to systemic immune dysregulation, which can impair wound healing, increase risk of infection, and potentially interfere with neuroregeneration [18]. Further exacerbating these issues is the fact that individuals with SCI often do not adhere to nutrition recommendations, which could worsen their GI complications [19,20,21].

Current bowel care programs, including medications and evacuation techniques, are tailored to symptom management of neurogenic bowel dysfunction focused on regular bowel movements and adequate stool consistency, rather than addressing the root of GI discomfort as well [22]. Probiotics, live microbes that add helpful strains to the gut microbiome, and prebiotics, which promote the growth of beneficial bacteria, hold potential as therapeutic avenues to restore gut microbiota balance after SCI [10,23,24]. Most positive findings regarding improved gut microbiota balance are from studies on non-disabled individuals. During probiotic supplementation, a relative higher abundance of the ingested strains in the gut can be observed [25,26,27,28]. Furthermore, anti-inflammatory effects of probiotics could be proven for various probiotic strains; the exact effect however is strain-dependent and can lead to an improved gut barrier, decreased intestinal permeability, and a decrease in GI symptoms [25,26,29,30]. Studies on probiotic effects on irritable bowel syndrome also showed improved GI symptoms alongside microbiome changes, suggesting a direct link, which could extend to other bowel diseases [31,32,33]. Currently, there is limited evidence available specific to SCI. A cross-over pilot study involving wheelchair athletes, of whom 11 had a SCI, demonstrated good tolerance of both probiotic and prebiotic supplementation [34]. Out of fourteen participants, only one reported minor side effects. Further analyses indicated a positive effect of probiotics on gut microbiome composition and inflammatory status [35], though there was no improvement in subjective GI health [34]. However, higher microbiome diversity has been shown in athletes compared to non-athletes [36,37,38], which may indicate lower inflammatory status and fewer GI symptoms in athletes. This could potentially blunt the effects of probiotics.

To accurately assess the potential of probiotic and prebiotic supplementation in alleviating GI discomfort, it is essential to conduct an intervention study in the broader population with SCI—particularly among those suffering from GI-related complications. The primary objective of this study is to evaluate the effect of probiotic and prebiotic supplementation in improving GI health in individuals with chronic SCI experiencing GI complaints, as measured by the Gastrointestinal Quality of Life Index (GIQLI). As secondary aims, further effects on GI health, including changes in gut microbiome composition, inflammatory serum markers, and GI transit time (GITT) will be assessed. Probiotic supplementation is hypothesized to improve GIQLI scores and decrease GITT and inflammatory serum markers, as well as increasing the relative abundance of beneficial microbial strains and decreasing inflammation-associated genera in the gut.

## 2. Materials and Methods

### 2.1. Study Design, Setting, and Sample Size

**Figure 1:**
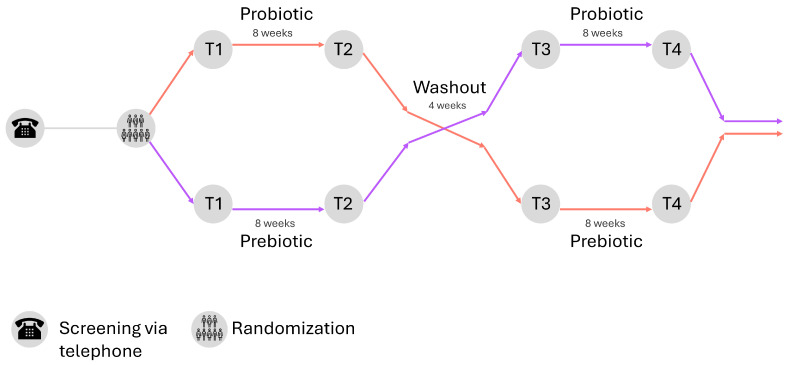
Summary of study design. Participants are randomized into a supplement group. Supplements have to be taken daily for eight weeks (T2), followed by a four-week washout (T3) and a crossover to the other intervention for another eight weeks (T4).

This is a single-center open-label two-arm randomized controlled crossover superiority trial (RCCT) adhering to CONSORT guidelines [39] with a total duration of 20 weeks (Supplementary Material). The study is conducted at the Swiss Paraplegic Research in Nottwil, Switzerland. It involves two 8-week interventions consisting of daily intake of either a probiotic (Biotics-G, Burgerstein AG, Rapperswil-Jona, Switzerland) or a prebiotic (Oat Bran, Naturaplan, manufactured by Swissmill, Zurich, Switzerland) supplement, separated by a 4-week washout period (Figure 1). Group allocation, which determines the order of supplement intake by the participants, is determined using a permuted block randomization (block size 4). For this, a randomization list was created using the statistical software Stata (Stata. Version 18. College Station, TX, USA: StataCorp, 2024). Due to the nature of the supplements, no allocation concealment or blinding of study personnel or participants is possible. Recruitment for this study started in summer 2025, and data collection will continue for two years; the study is therefore expected to end in summer 2027. The low-risk nature of this study warrants a risk-based approach for study monitoring. The study will be conducted in accordance with the Declaration of Helsinki. The study was approved by the Ethics Committee for Northwest/Central Switzerland (EKNZ, ID: 2025-00238, 24 February 2025, Version 2.0). The study is registered at ClinicalTrials.gov (ID: NCT06870331, 2 April 2025). Written informed consent will be obtained from all participants involved in the study.

The sample size was calculated for the primary outcome (changes in GIQLI) based on the results of a previous pilot study [34], applying a clinically relevant mean difference of 10 points on the GIQLI [40,41,42] and correcting for a larger expected variability in the GIQLI in the current study population. Assuming a significance level of 5% and a power of 85%, a sample size of 50 participants, with 25 participants in each group, was determined.

### 2.2. Study Participants

Adult (≥18 years old) male and female individuals with chronic (>1 year) traumatic and non-traumatic SCI, with a complete or incomplete lesion according to the American Spinal Injury Association Impairment Scale (AIS A-D), are invited to participate. Individuals experiencing significant GI complaints, indicated with ‘yes’ at screening and a GIQLI score below 110 at baseline (T1), are included. Exclusion criteria are antibiotic use, major dietary changes within 4 weeks before the study, presence of a diagnosed clinically relevant medical condition (e.g., Crohn’s disease, diagnosed eating or GI disorders), intake of significant concomitant medication (e.g., immunomodulating therapy, mesalazines), and pregnancy. No concurrent participation in other clinical trials investigating or potentially affecting GI health is allowed. Non-adherence to the supplementation, defined by missing >25% of supplement doses, can lead to exclusion from the trial at the investigator’s discretion.

Potential participants are recruited by distributing the study information and a flyer within the networks of the Swiss Paraplegic Association, the Swiss Paraplegic Centre, and the Swiss Paraplegic Research. In the case of a low recruitment rate, study information will also be spread through community platforms, both online and in magazines (e.g., Paracontact). Interested participants are contacted by phone or in person to perform the screening, where eligibility criteria are assessed, and the participant is informed about the study and what procedures it entails. Written participant information and an informed consent form are provided. After at least 24 h of consideration time, informed consent will be obtained from each participant.

### 2.3. Intervention

The probiotic intervention involves an 8-week supplementation with the commercially available probiotic BIOTICS-G (Burgerstein AG, Rapperswil-Jona, Switzerland). Participants take one sachet (2.5 g) daily, mixed into 50–100 mL of water 30 min before breakfast or dinner. Biotics-G supplies fourteen lactobacilli and bifidobacteria strains together with Saccharomyces boulardii, delivering 2.5 × 10^9^ CFU per sachet. The composition closely mirrors Bactosan^®^ Pro-FOS, which improved microbial diversity and reduced systemic inflammation in a crossover pilot study involving wheelchair athletes with spinal cord lesions [35]. Bactosan^®^ Pro-FOS is no longer on the Swiss market, which is why the alternative probiotic BIOTICS-G had to be chosen. The present trial extends that evidence to a community cohort.

The prebiotic intervention involves taking a prebiotic supplement for 8 weeks. The participants take 5 g of oat bran daily (Oat Bran, Naturaplan, manufactured by Swissmill, Zurich, Switzerland), mixed in with their usual breakfast or dinner.

The probiotic supplementation serves as the intervention of interest and the prebiotic supplementation as an active comparator, based on the literature and a pilot study [35,43].

Participants are instructed to maintain their usual training routine and diet, to minimize the chance of potential health modifications to be attributable to any factor other than the intervention with pre- or probiotics. Participants receive weekly reminders to take supplements and are asked to return empty packages to the study center after the intervention periods as a means of control of adherence to supplementation.

The first intervention phase is followed by a 4-week washout period to prevent carryover effects. Previous studies in non-disabled individuals have proven 1–2 weeks to be sufficient for effects of inulin-type fructans (prebiotics) to disappear after the intake has ceased [44]. To ensure adequate washout in our study population, the washout period is extended to four weeks.

### 2.4. Assessments

Outcome data are gathered at four timepoints: baseline (T1), after the first eight-week intervention phase (T2), after the four-week washout (T3), and after the second intervention phase (T4) - an overview can be found in Table 1. The primary endpoint is the GIQLI, indicating changes in subjective GI health [45]. The GIQLI is a questionnaire consisting of 36 questions assessing health-related quality of life in GI disorders, each on a scale from 0 to 4 [46]. It captures a person’s own perception of symptoms, functional limitations, and the psychosocial impact thereof. The GIQLI can range from a score of 0 to 144, with higher scores indicating better gut health and satisfaction with gut health.

Secondary endpoints cover gastrointestinal transit, systemic and mucosal biology, and safety. GITT is measured in the home setting with the Blue Dye Method, defined as the interval between ingestion of blue food coloring on an empty stomach and the first and last appearance of blue-colored stool [47,48]. Blood serum collected at each visit is analyzed with the Olink Target 48 Cytokine panel (Olink Proteomics AB, Uppsala, Sweden), which quantifies 45 inflammation-related proteins, including cytokines and chemokines such as IL-2 and TNF-α. These markers have been shown to be elevated in the serum of individuals with chronic SCI patients compared to healthy controls [49]. Fecal microbial community structure is characterized by full-length 16S rRNA gene sequencing on the MinION Nanopore sequencer (Oxford Nanopore Technologies, Oxford, UK). DNA is extracted with the QIAamp PowerFecal Pro DNA kit (QIAGEN, Hilden, Germany) using ten-minute bead-beating with 0.1 mm zirconia beads, quantified with Qubit™ 4.0 dsDNA HS fluorimeter (Thermo Fisher Scientific, Waltham, MA, USA), and prepared with the MinION Nanopore Ligation Sequencing Kit (SQK-LSK114) plus native barcodes (EXP-PBC096) (Oxford Nanopore Technologies, Oxford, UK). Libraries are pooled equimolarly, loaded on R10.4.1 flow cells, and base-called with Dorado; reads below a quality score of 9 are discarded. Emu assigns taxonomy. Supplement tolerance and all adverse events are documented throughout the study.

Variables that may influence these outcomes are captured systematically. At T1, each participant provides age, sex, lesion level and completeness, time since injury, body mass, and current medication (Appendix, Table A1) Physical activity is quantified with the Leisure-Time Physical Activity Questionnaire for persons with SCI (LTPAQ-SCI) [50], while autonomic function is evaluated with the ISAFSCI (second edition, 2021) tool [51]. Dietary intake is recorded with a three-day diary before every visit and processed in Prodi 7.4 (Version 7.4, Nutriscience, Stuttgart, Germany); mean energy, fiber, saturated fat and alcohol intakes are used in sensitivity analyses to mitigate dietary confounding.

Sample logistics follow a standardized pipeline. During the week preceding each visit, participants collect stool in the OMNIgene^®^ GUT (DNA Genotek, Ottawa ON, Canada) and draw 4.9 mL of venous blood into an S-Monovette Serum tube (Sarstedt, Sevelen, Switzerland). Blood is centrifuged within thirty minutes at 1800× *g*, 20 °C, for fifteen minutes. Plasma is aliquoted in 500 µL volumes and stored at −80 °C. Stool is vortexed on arrival, subdivided into 250 µL aliquots, and frozen at −80 °C. To align sampling with supplementation phases, T1 occurs one week before tablets or oat bran are first taken, providing sufficient time to complete home procedures. Thereafter, all home collections take place in the week before each center visit, so that the full protocol spans 21 weeks although active supplementation occupies 20 weeks. Participants receive weekly electronic reminders covering supplement intake, as well as the timing of stool, diary, and GITT procedures.

## 3. Data Analyses

An intention-to-treat analysis will be conducted to minimize potential bias resulting from disruption to baseline equivalence following randomization, which may occur due to participant withdrawal, non-adherence to the study protocol, or excessive missing data. Participants will be included in analyses only where data are available. Protocol deviations, early withdrawals, and loss to follow-up will be documented and evaluated. Basic univariable statistical analysis techniques will be used to describe the study population as well as the primary and secondary outcomes at the different measurement points. To evaluate the longitudinal variation in the primary outcome and secondary outcomes, we will use multilevel mixed-effects models that appropriately account for the within- and between-individual sources of variance in outcome variation. In these models, participants are treated as random effects, while intervention and period are used as fixed effects. All models will be adjusted for the baseline value of the respective outcome measured before each period. To evaluate potential carryover effects, sequence (AB vs. BA) will be included as a fixed effect, and treatment-by-period interactions will be tested. Furthermore, pre-period baselines will be compared to verify that the gut environment returned to its SCI-baseline during washout, and a carryover term will be incorporated into the model to identify residual effects. If significant carryover is detected, a sensitivity analysis using only period 1 data will be performed to provide an unbiased estimate of the intervention effect. Depending on the findings, subgroup analyses may be performed for personal parameters (e.g., sex, lesion level). For the microbiome analyses, diversity indices will be compared using paired *t*-tests while correcting for multiple testing using the Benjamini–Hochberg method. A significance level of 5% will be applied. Protein concentrations will be reported as absolute abundances in pg/mL. Absolute abundance will be tested with mixed-effects models analogous to the primary analysis. The Benjamini–Hochberg procedure will control the false-discovery rate at 5%. Multi-omics integration will employ sparse partial least-squares regression to relate inflammatory proteins, microbial genera, and clinical outcomes (GIQLI, GITT). Statistical software packages to be used include R (Version 4.5.2), Stata (Version 19.0), and SPSS (Version 31.0), or any newer stable version available at the time of data analysis.

### Patient and Public Involvement Statement

There was no involvement of patients or the public in the design, conduct, reporting, or dissemination planning of this trial. However, the research addresses priorities identified by individuals with SCI, particularly bowel management and discomfort in daily life [4,6]. Findings will be shared with the community in appropriate formats, online, and in magazines.

## 4. Strengths and Limitations

GI dysfunction is a highly prevalent and burdensome complication in SCI, often resulting in reduced quality of life and compromised autonomy. Despite its significance, effective and targeted therapeutic strategies remain limited. This study presents a unique approach by utilizing pro- and prebiotics to target the microbiome and gut dysbiosis. This study employs a crossover design, which is particularly advantageous in controlling for inter-individual variability, a known confounding factor in microbiome-related research [52,53,54,55]. The clear strength of the crossover design is that each participant acts as his own control by receiving both supplements. Further, statistical power is higher, and the required sample size to detect meaningful effects is lower compared to a parallel-group RCT [56]. An additional strength is the concurrent profiling of the fecal microbiome and 45 circulating inflammatory proteins, which supports mechanistic interpretation of clinical findings. The standardized processing pipeline and inclusion of internal controls enhance analytical reproducibility. A similar protocol implemented in wheelchair athletes, of whom the majority had a SCI, demonstrated its feasibility in this population [34].

The combination of subjective and objective outcome measures ensures meaningful findings to be detected and to hold translational relevance. The usage of commercially available supplements enhances the potential for a real-world, feasible intervention in clinical and community-based settings, outside of research, and increases the scalability of the findings. Based on previous experience in the pilot study, a sample size of 50 is realistically achievable.

The study is subject to several limitations. The open-label nature of the study might introduce a potential bias. With the probiotic packaging resembling a medicinal product, versus the prebiotic clearly coming from a grocery store, participants may have different expectations for the efficacy of either supplement, which may bias their responses. This is not preventable, given the need for specific packaging for both supplements. Additionally, as diet and lifestyle are not strictly controlled, microbiome changes may be confounded, which in a real-life setting cannot be avoided. Despite not being controlled, by assessing physical activity and nutrition at each timepoint, big lifestyle changes can be detected and taken into consideration when analyzing the data. Further, at home supplementation makes strict control of adherence impossible; however, by asking participants to return empty packages after the intervention period, a sense of control is added and quantification of adherence is possible.

Another potential limitation of this crossover design is the risk of carryover effects, where the physiological and microbial changes from the first intervention might persist into the second period. Given the neurogenic bowel dysfunction inherent in SCI, transit times are prolonged, which could theoretically extend the influence of interventions. However, this risk is mitigated by the implementation of a 4-week washout period, exceeding the 2-week window typically required for the microbiome to revert to baseline after a dietary perturbation, and by employing a rigorous four-tiered statistical framework to detect sequence and period interactions [57,58,59,60].

## 5. Conclusions and Outlook

With GI discomfort and bowel management being priorities of the community with SCI, and the high numbers of affected people within the community, studies like this are very much needed. Both probiotic and prebiotic supplementation will be evaluated as potential treatment avenues for GI discomfort and gut dysbiosis. The effects will be measured by both patient-reported outcomes, e.g., GIQLI, and objective measures, e.g., microbiome composition, blood inflammation markers, and GITT, and we aim to assess changes in gut health and gut health-related quality of life. The inclusion of subjective and objective measures enhances translational relevance.

Compared to existing research, this study advances the field by addressing an underexplored intersection between microbiome science, GI health, and quality of life in SCI. Findings from this study will be shared with the clinic and the community with SCI to allow for more informed consulting of patients with SCI and GI dysfunction. This may lower the burden of bowel problems in SCI in the long-term and improve experienced quality of life for affected persons.

## Figures and Tables

**Table 1:** Overview of assessments and data collection at the measurement timepoints: T1 (baseline), T2 (after first intervention), T3 (after washout), T4 (after second intervention).

	T1	T2	T3	T4
Timepoint	Week 1	Week 8	Week 12	Week 20
Location	Study center	Study center	Study center	Study center
Sociodemographic Characteristics 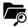	X			
Changes in Diet and Medication 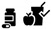	X	X	X	X
GIQLI 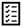	X	X	X	X
LTPAQ-SCI 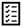	X	X	X	X
ISAFSCI 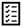	X	X	X	X
Blood Sample 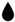	X	X	X	X
Location	At home	At home	At home	At home
Stool Sample 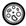	X	X	X	X
GITT 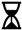	X	X	X	X
3-Day Food Diary 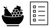	X	X	X	X

Note: GIQLI = Gastrointestinal Quality of Life Index, GITT = gastrointestinal transit time, ISAFCSI = International Standards to Document Remaining Autonomic Function after Spinal Cord Injury, LTPAQ-SCI = Leisure Time Physical Activity Questionnaire for persons with spinal cord injury.

## Data Availability

In this study protocol, no data was collected or analyzed. Data sharing thus does not apply to this article.

## Supplementary Materials

The following supporting information can be downloaded at: https://www.mdpi.com/article/10.3390/mps9010014/s1, Figure S1: CONSORT Flowchart PRO-GIDSCI.

## Abbreviations

The following abbreviations are used in this manuscript:

AIS	American Spinal Injury Association Impairment Scale
CFU	Colony Forming Units
GIQLI	Gastrointestinal Quality of Life Index
GI	Gastrointestinal
GITT	Gastrointestinal Transit Time
ISAFSCI	International Standards to Document Remaining Autonomic Function After Spinal Cord Injury
LTPAQ	Leisure Time Physical Activity Questionnaire
RCCT	Randomized Controlled Crossover Trial
SCI	Spinal Cord Injury

## Appendix A

**Table A1:** Overview of sociodemographic data collected at each timepoint. AIS Score = American Spinal Injury Association Impairment Scale, SCI = spinal cord injury, GI = gastrointestinal.

Variable	Specification	Timepoint
Year of Birth		Screening
Sex	Female: currently pregnant?	Screening
Date of SCI		Screening
AIS Score		Screening
Antibiotics	Yes/no during the past 4 weeks	Screening
Height		T1
Body Mass		T1, T2, T3, T4
Cause of SCI	Traumatic or non-traumatic	T1
Lesion Level		T1
GI Disease		Screening, T1
Further Diseases		T1
Medication Intake		Screening, T1, T2, T3, T4
Diet	Normal, vegetarian, vegan, etc.	Screening, T1, T2, T3, T4
Sports	Sport type and hours training per week	T1
