# Supplementary figures and images for: Probiotic and Prebiotic Supplementation for Gastrointestinal Discomfort in Chronic Spinal Cord Injury (PRO-GIDSCI): A Randomized Controlled Crossover Trial Protocol
Source: Methods Protoc. 2026 Jan 17;9(1):14. doi: 10.3390/mps9010014 (PMC12821591; doi:10.3390/mps9010014)

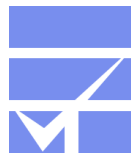

# CONSORT

## TRANSPARENT REPORTING of TRIALS

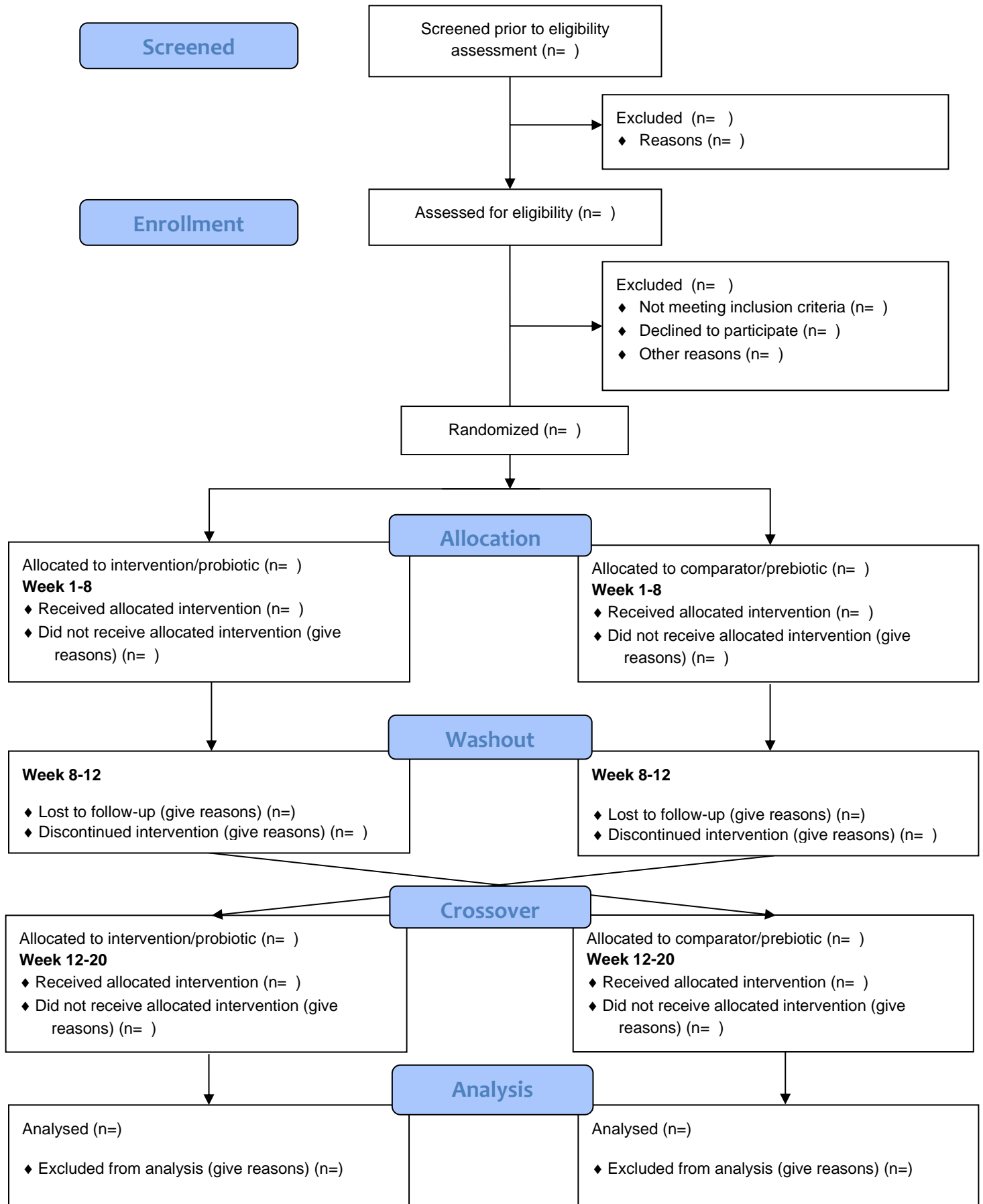

Supplement: Supplementary file 1 [file mps-09-00014-s001.zip › mps-3976006-supplementary.pdf]
